# A multimodal machine learning model for predicting postoperative worsening of FOGQ in Parkinson’s disease following STN-DBS

**DOI:** 10.3389/fneur.2026.1767807

**Published:** 2026-05-05

**Authors:** Min Xu, Shuhong Mei, Shuming Huang, Longyuan Gu, Yuting Zhang, Siyan Chen, Yuyao Tian, Li Du, Hui Zhao, Zixuan Zhang, Ruyi Chen, Guiyun Cui, Wei Zhang, Jie Zu

**Affiliations:** 1 Department of Neurology, Parkinson’s Disease Center, The Affiliated Hospital of Xuzhou Medical University, Xuzhou, China; 2 Department of Neurology, The First Clinical College, Xuzhou Medical University, Xuzhou, China; 3 Department of Neurosurgery, Ji'an Central People's Hospital, Ji'an, Jiangxi, China; 4 Department of Biomedical Engineering, The Chinese University of Hong Kong, Hong Kong, Hong Kong SAR, China

**Keywords:** deep brain stimulation, freezing of gait, machine learning, Parkinson’s disease, prediction model

## Abstract

**Objective:**

To develop and validate a multimodal machine learning model to predict postoperative worsening of freezing of gait questionnaire (FOGQ) scores in patients with Parkinson’s disease (PD) undergoing subthalamic nucleus deep brain stimulation (STN-DBS).

**Methods:**

This retrospective study analyzed data from 134 patients with PD who underwent bilateral STN-DBS. The model integrated four data modalities: clinical scale assessments, structural neuroimaging features derived from voxel-based morphometry (VBM), stereotactic electrode localization data via Lead-DBS analysis, and radiomics features extracted from preoperative MRI. Following standardization, feature selection was conducted using LASSO, Boruta and recursive feature elimination with cross-validation (RFECV) methods to identify the most relevant predictors. Multiple machine learning algorithms were evaluated. Model development and internal validation were conducted using a 5-fold nested cross-validation framework. Model performance was assessed using ROC curves, calibration curves, and decision curve analysis, and model interpretability was analyzed using SHAP values.

**Results:**

The LightGBM model achieved the highest AUC of 0.917 for predicting FOGQ deterioration. The analysis emphasized the importance of multimodal data integration, combining clinical, structural, and radiomic features to enhance predictive accuracy.

**Conclusion:**

This multimodal LightGBM model achieved robust discrimination between patients with and without postoperative FOGQ deterioration, highlighting the value of integrating clinical, structural, and radiomic features for preoperative risk stratification in PD patients undergoing STN-DBS. These findings may inform personalized patient selection, early identification of high-risk individuals, and treatment planning, though external validation in prospective multicenter cohorts remains a necessary next step.

## 1 Introduction

Parkinson’s disease (PD) is the second most prevalent neurodegenerative disorder, marked by a constellation of motor and non-motor symptoms that profoundly impair patients’ quality of life. Freezing of gait (FOG) is one of the most disabling symptoms of PD, and patients with FOG are prone to falls, fractures, reduced quality of life, and loss of independence. Understanding and treating gait disorders and FOG represent urgent, high-priority needs and were designated the foremost research priorities at the 2014 International Parkinson and Movement Disorder Society meeting (1). However, the pathophysiology of FOG remains incompletely understood and likely involves distributed cortical, basal ganglia, brainstem, and cerebellar circuits (2–4). Deep brain stimulation of the subthalamic nucleus (STN-DBS) is an established treatment for the motor symptoms of advanced PD (5). However, the extent of symptom relief varies markedly between individuals, particularly for axial features such as FOG. Some patients may even experience worsening of FOG (6–8). This highlights the importance and complexity of patient selection and evaluation prior to surgery, necessitating more accurate and convenient prediction models to assess the postoperative FOG outcomes in patients before the surgery.

Previous studies have employed wearable devices and gait biomechanical parameters, in conjunction with gait characteristics such as stride length, walking speed, and gait stability, to predict the occurrence of FOG in PD (9, 10). Although these studies have enabled early detection through gait analysis, variability in wearable-device protocols and the high cost of such equipment hinder widespread adoption. Moreover, evidence linking specific gait metrics, including stability related measures, to FOG severity and underlying mechanisms remains limited and heterogeneous (11, 12). In contrast, neuroimaging biomarkers such as voxel-based morphometry (VBM) and surface-based morphometry (SBM) can robustly quantify cortical morphology and have been shown to predict PD progression (13, 14). More recently, radiomic analysis of MRI has been applied to differentiate PD subtypes and to forecast cognitive decline (15, 16), highlighting the potential of high-dimensional image-derived features to capture subtle microstructural changes. Neuroimaging- and connectome-based models have used PET biomarkers, fMRI connectivity signatures, and T1-weighted structural measures to predict STN-DBS outcomes, but most focus on overall motor benefit rather than gait-related outcomes (17–20). Machine learning frameworks are well suited to integrate such imaging features with clinical data and have already been used to predict optimal DBS parameters and postoperative quality-of-life changes in PD (21, 22). However, in contrast to a recently published study on postoperative FOG after STN-DBS (23), we leveraged multimodal data and treated the STN, the principal DBS target, as the primary region of interest (ROI) to predict postoperative worsening of FOG in patients with PD undergoing STN-DBS.

Against this background, the present study sought to develop a multimodal machine learning model to predict postoperative FOG deterioration after STN-DBS in PD. To this end, we integrated preoperative clinical measures, voxel-based structural MRI indices, radiomic features extracted from T1- and T2-weighted images of the STN, and electrode localization within functionally defined STN subregions into a unified predictive framework. We hypothesized that this multimodal, STN-centered approach would improve discrimination between patients with and without postoperative FOG worsening and could provide a framework to support preoperative risk stratification, individualized target planning, and postoperative management, pending external validation.

## 2 Materials and methods

### 2.1 Research subjects

This retrospective cohort initially comprised 253 patients with PD who underwent bilateral STN-DBS at the Department of Neurology, Affiliated Hospital of Xuzhou Medical University, between September 2018 and February 2023. The research protocol was approved by the hospital ethics committee (XYFY2023-KL422-01). Inclusion criteria were: (1) Age ≥18 years; (2) Definitive diagnosis of Parkinson’s disease according to the UK Parkinson’s Disease Society Brain Bank Clinical Diagnostic Criteria, validated by board-certified neurologists. Exclusion criteria were: (1) Evidence of structural brain lesions from traumatic injury, cerebrovascular events (infarction/hemorrhage), vascular malformations, or neoplastic processes; (2) History of intracranial surgical interventions (including endovascular or craniotomy procedures); (3) postoperative follow-up shorter than 24 months after DBS implantation or with incomplete follow-up documentation; (4) Concurrent disease-modifying therapies or clinical deterioration necessitating alternative interventions within 24 months postoperatively. In total, 119 patients were excluded owing to incomplete clinical, imaging, or follow-up data (*n* = 93), stimulation retargeting within 2 years of surgery (*n* = 5), or marked disease progression, defined as an increase in Hoehn-Yahr stage > 1 during follow-up (*n* = 21). The final cohort therefore comprised 134 patients.

Clinical and demographic data were obtained from the hospital electronic medical record system and standardized follow-up questionnaires, including age, sex, disease duration, and modified H-Y stage. Standardized assessments were conducted at two time points: preoperative baseline and 24 months after STN-DBS. Medication-OFF evaluations were performed after a 12-h levodopa withdrawal, and medication-ON evaluations were performed 60–90 min after administration of the optimized levodopa equivalent daily dose (LEDD). Motor severity was assessed using the UPDRS-III in both medication-OFF and medication-ON states preoperatively, and at the 24-month follow-up in the medication-OFF/DBS-ON condition. FOG severity was assessed using the FOGQ in the medication-OFF state preoperatively and under the same standardized condition at follow-up, maximizing comparability with the preoperative baseline. Accordingly, the present model predicts postoperative worsening of FOGQ scores under standardized OFF-medication/DBS-ON assessment conditions. ΔFOGQ was defined as the preoperative score minus the postoperative score (ΔFOGQ = Pre_score_—Post_score_). In the VBM analysis, we used the raw change in FOGQ (ΔFOGQ = Pre_score_—Post_score_) to examine the relationship between gray matter volume and the magnitude of postoperative FOGQ deterioration. This raw change score was used to directly investigate the association between anatomical changes and the observed FOGQ changes.

However, for the primary prediction model, we used the baseline-normalized change score (ΔFOGQ_norm_) to reduce potential biases due to differing baseline severities across patients. The primary outcome for model development was the dichotomized postoperative FOGQ status (deterioration vs. non-deterioration). Change was quantified using the baseline-normalized FOGQ change (*Δ*FOGQ_norm_), calculated as: $\;\text{ΔFOG}Q_{\mathrm{norm}}=\frac{\mathrm{FOG}Q_{\mathrm{pre}}-\mathrm{FOG}Q_{\text{post}}}{\mathrm{FOG}Q_{\max}-\mathrm{FOG}Q_{\mathrm{pre}}}$ . FOGQ_pre_ < FOGQ_max_ for all patients; therefore, the denominator was non-zero in all cases. Deterioration was defined as ΔFOGQ_norm_ < 0, and non-deterioration as ΔFOGQ_norm_ ≥ 0. We chose the two-year postoperative time point as the primary follow-up endpoint, as this protocol-defined evaluation window coincides with the neuroplasticity-driven stabilization phase of STN-DBS effects (24–26). Moreover, we recognize that disease progression may affect the scores. To limit confounding by disease progression, we included patients with relatively stable clinical status; baseline H-Y stage was homogeneous and did not worsen by more than one stage during follow-up. The cohort selection flowchart is shown in Supplementary Figure 1.

### 2.2 Neurosurgical procedures

All patients underwent bilateral STN-DBS performed by the same multidisciplinary team using a standardized stereotactic protocol (27). Preoperative MRI and CT images were fused in the surgical planning system to calculate the coordinates and design the trajectories of the bilateral STN. Under local anesthesia, a Leksell stereotactic frame was applied, and microelectrode recordings were used to refine targeting before implantation of Medtronic 3,389 leads. In a second stage performed under general anesthesia, the extension cables and an implantable pulse generator (Activa PC or Activa RC, Medtronic) were implanted. Postoperative CT within 1–3 days confirmed lead position and excluded intracranial hemorrhage.

### 2.3 Image acquisition

All preoperative MRI scans were acquired within 24 h before STN-DBS on a 3.0 T scanner (DISCOVERY MR750w, GE Healthcare) using a 24-channel head coil. High-resolution 3D T1-weighted images were acquired with a BRAVO sequence (1 × 1 × 1 mm^3^; 156 slices; TR/TE/TI = 8.644/3.216/450 ms; flip angle = 12°; FOV = 256 × 256 mm^2^). Axial 2D T2-weighted FSE images were acquired (TR/TE = 5241/105.4 ms; 2.0-mm slices, no gap; 48 slices; matrix = 512 × 512; FOV = 240 × 240 mm^2^; NEX = 3).

### 2.4 Structural brain image preprocessing and feature extraction

Structural T1 images were processed in SPM12/CAT12 (MATLAB R2022a) using a standard VBM pipeline (28, 29). Images were segmented and normalized to MNI space to derive gray matter volume (GMV) maps. Voxel-wise group differences were tested using a GLM with age, sex, and total intracranial volume (TIV) as covariates. Multiple comparisons were controlled using GRF correction (two-tailed; voxel-level *p* < 0.001, cluster-level *p* < 0.05). For descriptive purposes, the whole-brain VBM group comparison was performed at the cohort level and was not used to define or select predictive features. All GMV predictors used for machine learning were extracted from *a priori* atlas-defined anatomical ROIs (AAL3), independent of any VBM statistical maps or significant clusters.

### 2.5 Radiomics feature processing

All MRI data were converted to NIfTI format, anonymized, and preprocessed with N4 bias field correction, resampling to 1 × 1 × 1 mm^3^ isotropic voxels, and intensity normalization. T1-weighted images were rigidly coregistered to the corresponding T2-weighted images using Advanced Normalization Tools (ANTs).

The STN was defined as the ROI. For each patient, STN masks were manually delineated on preoperative T2-weighted images in ITK-SNAP and propagated to the corresponding T1- and T2-weighted images. Using the PyRadiomics package, 944 features were extracted from each STN ROI, including first-order statistics, shape descriptors, and higher-order texture features derived from original, Laplacian-of-Gaussian-filtered, and wavelet-transformed images.

To assess reproducibility, 30 patients were randomly selected for a second, independent STN segmentation and feature extraction (30, 31). Intraclass correlation coefficients (ICC) were calculated for all features, and those with ICC < 0.75 were excluded as a label-free quality-control prefilter. Subsequent radiomics feature-selection steps and construction of the composite Rad-score are described in Section 3.4.

### 2.6 Electrode target points and subregion locations

The Lead-DBS toolbox in MATLAB was used to localize intracranial electrode contacts after DBS surgery (32). Preoperative MRI and postoperative CT DICOM images were imported, coregistered with ANTs and SPM, and normalized to MNI space. DBS leads were reconstructed using the PaCER or refined TRAC/CORE algorithm, and the spatial relationship between active contacts and subcortical structures (STN, GPe, GPi, and red nucleus) was visualized using the DISTAL atlas. In this atlas, the STN is parcellated into motor, associative, and limbic subregions; in the present study, these were further merged into motor and non-motor territories. For each hemisphere, we coded whether the active contact overlapped the motor or non-motor STN, and this categorical variable was included as an imaging feature in the prediction models. Illustrative examples of STN segmentation and electrode localization are shown in Supplementary Figure 2.

### 2.7 Multimodal feature selection

Across the nested cross-validation, all feature selection and hyperparameter tuning steps were performed using training data only within each resampling step, and the held-out outer test fold was used exclusively for final evaluation. To integrate multimodal information and reduce feature dimensionality, we used a three-step feature-selection procedure, with the following details: Stage 1, LASSO logistic regression (L1-regularized) was applied to the standardized multimodal feature pool, The regularization strength (C) was selected via stratified internal cross-validation using ROC-AUC. L1 regularization encourages sparsity in the model, leading to the selection of non-zero features. The solver used was liblinear, an optimization algorithm suitable for linear models with L1 regularization, with the max_iter parameter set to 5,000 to ensure convergence. Stage 2, A Boruta-style feature selection was applied using a RandomForestClassifier (n_estimators = 300, class_weight = balanced), which compares real features against permuted shadow features across iterations. Features that consistently outperformed the shadow baseline were retained. This step helps identify the most important features by considering the feature importance across multiple iterations. Stage 3, Recursive feature elimination with cross-validation (RFECV) was applied to the Boruta-filtered set, using a logistic regression estimator with L2 regularization (penalty = L2, solver = liblinear, max_iter = 5,000) and ROC-AUC scoring. RFECV iteratively removes the least informative variables and the final subset is determined by cross-validation-based ROC-AUC. When the candidate set was too small for RFECV, all candidates were retained. To ensure consistency in model input dimensionality and avoid overfitting due to small sample sizes, we constrained the number of predictors entering model fitting to eight within each outer training fold. If RFECV returned more than eight features, we truncated the set to the top eight features based on fold-specific importance/ranking. If fewer than eight features were selected, we supplemented the set to eight by adding features from the preceding candidate pool based on fold-specific ranking. Taken together, LASSO, Boruta, and RFECV played complementary roles: LASSO and Boruta provided a robust coarse screening of the high-dimensional multimodal space, whereas RFECV further refined this candidate pool to a compact and stable subset (fixed-size within each outer fold) of highly informative clinical and imaging predictors.

### 2.8 Model development and validation

**Figure 1:**
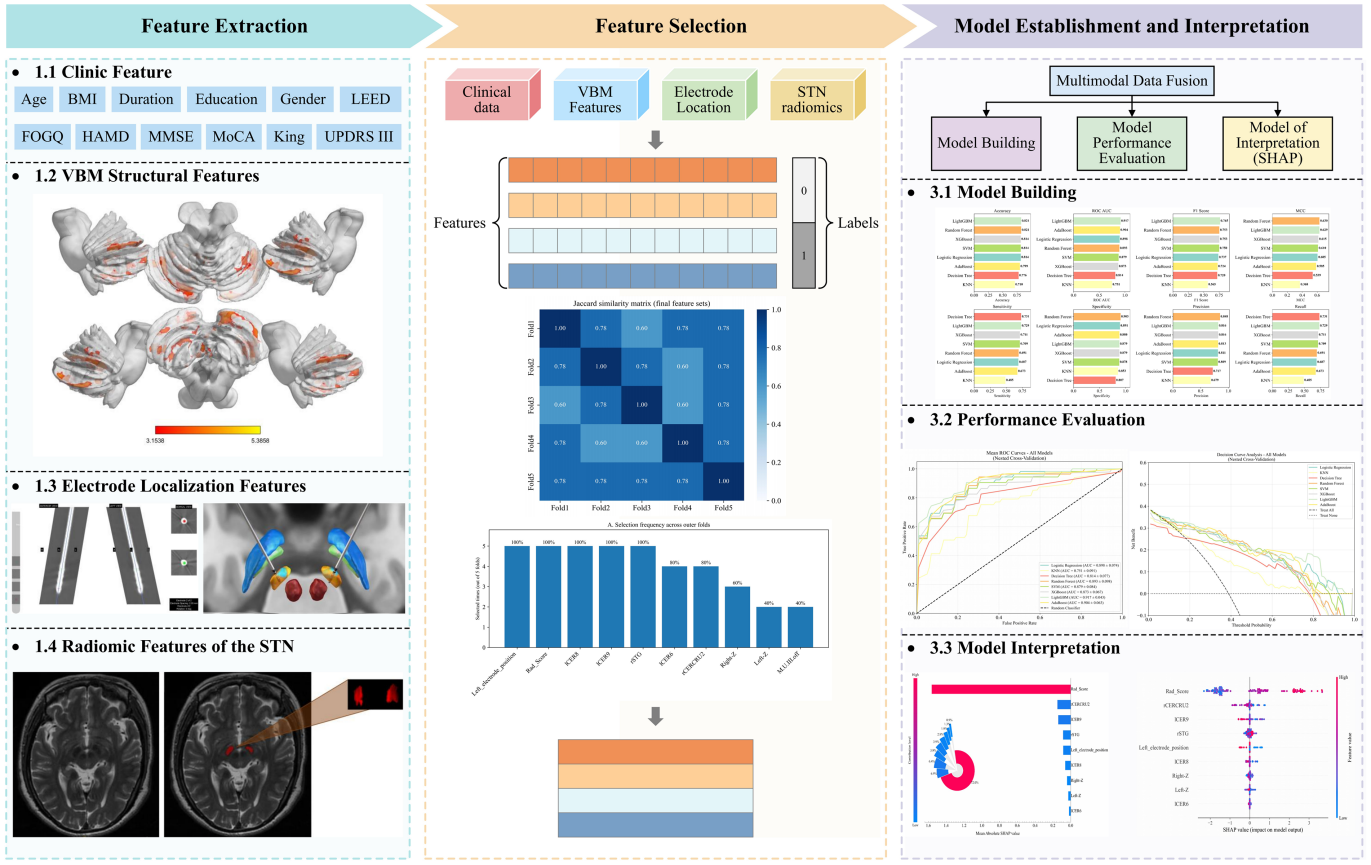
Workflow of the multimodal machine-learning pipeline for predicting postoperative worsening of FOGQ after STN-DBS. Preoperative clinical variables, VBM-derived gray-matter volumes, electrode-localization features (Lead-DBS), and STN radiomic features from T1- and T2-weighted MRI were extracted and fused into a multimodal feature set. Within a 5-fold nested cross-validation framework, features were selected in each outer training fold using LASSO, Boruta, and RFECV, and then used to train multiple classifiers. Model performance was evaluated on the held-out outer test folds, and SHAP was applied to interpret the best-performing LightGBM model and quantify the contribution of each feature group.

An overview of the multimodal machine-learning pipeline for predicting postoperative FOGQ deterioration is summarized in Figure 1. To avoid optimistic bias and information leakage, we adopted a 5-fold nested cross-validation (CV) framework for model development and internal validation. In the outer loop, the full cohort was randomly partitioned into five approximately equal folds, stratified by postoperative FOGQ outcome. In each iteration, four folds were used as the outer training set and the remaining fold served as the outer test set to obtain an unbiased performance estimate.

Within each outer training set, the three-stage feature-selection procedure (LASSO, Boruta, and RFECV) was performed using the outer training data only (with internal cross-validation where applicable), the number of predictors entering model fitting was constrained to eight within each outer fold, and an inner 5-fold CV loop within the outer training set was used for feature selection and hyperparameter tuning. All preprocessing steps—including z-score standardization of continuous variables, one-hot encoding of categorical variables, and the multimodal feature-selection pipeline combining LASSO, Boruta and RFECV—were fitted using training data only within the resampling framework and then applied to the corresponding validation folds, thereby preventing any information from the outer test fold from influencing model construction. For each algorithm, candidate hyperparameters were tuned to maximize the mean inner-loop area under the receiver operating characteristic curve (AUC).

The optimized hyperparameters were then used to refit each model on the entire outer training set, and performance was evaluated on the corresponding outer test fold. This procedure was repeated across all five outer folds, yielding five independent performance estimates for each algorithm. For each outer test fold, evaluation metrics (AUC, accuracy, sensitivity, specificity) were calculated separately, and results were reported as mean ± standard deviation (SD) across the five outer folds. Confusion matrices were computed for each outer test fold and averaged across outer folds to summarize the distribution of true positives, true negatives, false positives, and false negatives.

To evaluate the potential clinical utility of the prediction models, we performed decision curve analysis (DCA), plotting net benefit across a range of threshold probabilities and comparing each model with the “treat-all” and “treat-none” strategies. In each outer fold, SHAP values were computed on the held-out test set using the model trained on the corresponding outer training set with the fold-specific eight-feature set, with background data drawn from the outer training set. Global feature importance was summarized by aggregating mean absolute SHAP values across outer folds for each variable, and representative SHAP summary and dependence plots are presented in the Results.

### 2.9 Statistical analysis

All statistical analyses in this study were conducted using Python (version 3.10) and R software (version 4.2.2). For the descriptive statistics of baseline characteristics, categorical variables were presented as frequency and percentage (n, %), and intergroup comparisons were made using Pearson’s chi-squared test. Continuous variables were presented as mean ± SD or median (IQR), as appropriate. For normally distributed continuous variables, the Welch two-sample t-test was used for comparisons, while the Wilcoxon rank sum test was applied for non-normally distributed variables. A *p*-value < 0.05 was considered statistically significant. Univariable and multivariable logistic regression analyses were performed for postoperative FOGQ deterioration. Variables with *p* < 0.05 in univariable analyses were entered into the multivariable model, with baseline FOGQ forced in to mitigate baseline coupling. Adjusted odds ratios (aORs) with 95% confidence intervals (CIs) were reported. As a post-hoc exploratory analysis, Spearman rank correlation was used to assess the associations between ΔFOGQ and postoperative LEDD reduction percentage and stimulation frequency.

## 3 Results

### 3.1 Participants, baseline characteristics

Baseline characteristics of the 134 included patients (FOGQ non-deterioration, n = 82; deterioration, n = 52) are summarized in Table 1. Compared with the non-deterioration group, patients with postoperative FOGQ worsening were older at the time of surgery, had lower MoCA scores, higher UPDRS-III scores and were less likely to have electrodes targeting the STN motor subregion, particularly on the left side. At 24 months, overall DBS motor benefit was preserved (M. U. III.imp: 0.57 ± 0.13) and did not differ between groups (0.56 ± 0.12 vs. 0.59 ± 0.14; *p* = 0.166), suggesting that FOGQ worsening was unlikely due to ineffective STN-DBS. Disease duration, BMI, LEDD, and baseline H-Y stage did not differ significantly between groups.

**Table 1:** Demographic and clinical characteristics of all subjects.

Characteristic	Overall *N* = 134	Non-deterioration group *N* = 82	Deterioration *N* = 52	*p*-value
Gender				0.342
Male	73 (54.48%)	42 (51.22%)	31 (59.62%)	
Female	61 (45.52%)	40 (48.78%)	21 (40.38%)	
Age at surgery	64 (57, 69)	63 (55, 67)	66 (58, 69)	0.032
Duration	8.0 (6.0, 10.8)	8.0 (6.0, 10.0)	8.0 (6.0, 14.0)	0.334
BMI	23.9 (20.7, 26.0)	24.1 (20.8, 26.1)	23.4 (20.7, 25.6)	0.770
H-Y stage				0.422
<3	46 (34.33%)	26 (31.70%)	20 (38.46%)	
≥3	88 (65.67%)	56 (68.30%)	32 (61.54%)	
Residence				0.752
Rural	77 (57.46%)	48 (58.54%)	29 (55.77%)	
Urban	57 (42.54%)	34 (41.46%)	23 (44.23%)	
Education				0.366
Illiterate	22 (16.42%)	12 (14.63%)	10 (19.23%)	
Primary	27 (20.15%)	21 (25.61%)	6 (11.54%)	
Junior	47 (35.07%)	27 (32.93%)	20 (38.46%)	
Senior	17 (12.69%)	9 (10.98%)	8 (15.38%)	
University	21 (15.67%)	13 (15.85%)	8 (15.38%)	
LEDD	750 (563, 950)	750 (600, 900)	812 (500, 1,075)	0.340
FOGQ	12 (8, 17)	13 (10, 17)	10 (5, 13)	0.002
M.U.III.off	61 (45, 79)	57 (43, 75)	62 (54, 83)	0.099
M.U.III.on	28 (20, 38)	25 (20, 36)	31 (22, 43)	0.026
M.U.III.imp	0.52 ± 0.14	0.54 ± 0.15	0.50 ± 0.13	0.154
M.U.III.imp (Med-OFF/DBS-ON)	0.57 ± 0.13	0.59 ± 0.14	0.56 ± 0.12	0.166
NMSS	66 (33, 109)	70 (44, 107)	60 (26, 114)	0.326
King	20 (4, 42)	22 (4, 60)	20 (0, 32)	0.445
HAMA-14	16 (11, 21)	16 (11, 22)	16 (12, 20)	0.933
HAMD-24	21 (14, 27)	22 (16, 30)	19 (13, 26)	0.079
BDI	19 (11, 27)	20 (11, 28)	17 (11, 24)	0.270
MMSE	26.0 (23.0, 28.0)	26.0 (24.0, 28.0)	25.0 (21.0, 28.0)	0.166
MoCA	20.0 (16.3, 23.0)	21.0 (18.0, 24.0)	19.0 (14.8, 23.0)	0.024
Right electrode position				0.011
Motor area	70 (52.24%)	50 (60.98%)	20 (38.46%)	
Non-motor area	64 (47.76%)	32 (39.02%)	32 (61.54%)	
Left electrode position				<0.001
Motor area	66 (49.25%)	50 (60.98%)	16 (30.77%)	
Non-motor area	68 (50.75%)	32 (39.02%)	36 (69.23%)	

n (%); Median (IQR); Mean ± SD. H-Y stage, Hoehn and Yahr stage; LEDD, levodopa-equivalent daily dose; FOGQ, Freezing of Gait Questionnaire; M. U. III, Movement Disorder Society Unified Parkinson’s Disease Rating Scale Part III; NMSS, Non-Motor Symptoms Scale; King, King’s Parkinson’s Disease Pain Scale; HAMD-24, Hamilton Depression Rating Scale (24 items); HAMA-14, Hamilton Anxiety Rating Scale (14 items); BDI, Beck Depression Inventory; MMSE, Mini-Mental State Examination; MoCA, Montreal Cognitive Assessment.

### 3.2 Clinical data analysis

Univariate analyses showed no significant group differences in most baseline characteristics (*p* > 0.05). By contrast, several variables were significantly associated with FOGQ deterioration: older age at surgery (OR = 1.05, 95% CI: 1.00–1.09, *p* = 0.038), higher M. U. III.off scores (OR = 1.02, 95% CI: 1.00–1.03, *p* = 0.039), higher M. U. III.on scores (OR = 1.03, 95% CI: 1.00–1.06, *p* = 0.025), and lower MoCA scores (OR = 0.92, 95% CI: 0.86–0.99, *p* = 0.018). Motor-subregion targeting was also protective, for both the right electrode (OR = 0.40, 95% CI: 0.20–0.82, *p* = 0.012) and the left electrode (OR = 0.28, 95% CI: 0.14–0.59, *p* < 0.001).

In multivariable logistic regression with baseline FOGQ included as a covariate, three variables remained independently associated with postoperative FOGQ deterioration: higher M. U. III.on scores (aOR = 1.05, 95% CI: 1.01–1.08, *p* = 0.006), lower MoCA scores (aOR = 0.91, 95% CI: 0.84–0.99, *p* = 0.031), and motor-subregion targeting of the left electrode (aOR = 0.31, 95% CI: 0.14–0.66, *p* = 0.002), whereas other candidate variables did not reach significance in the fully adjusted model.

### 3.3 Voxel-based morphometry: brain structural differences associated with freezing of gait deterioration

**Figure 2:**
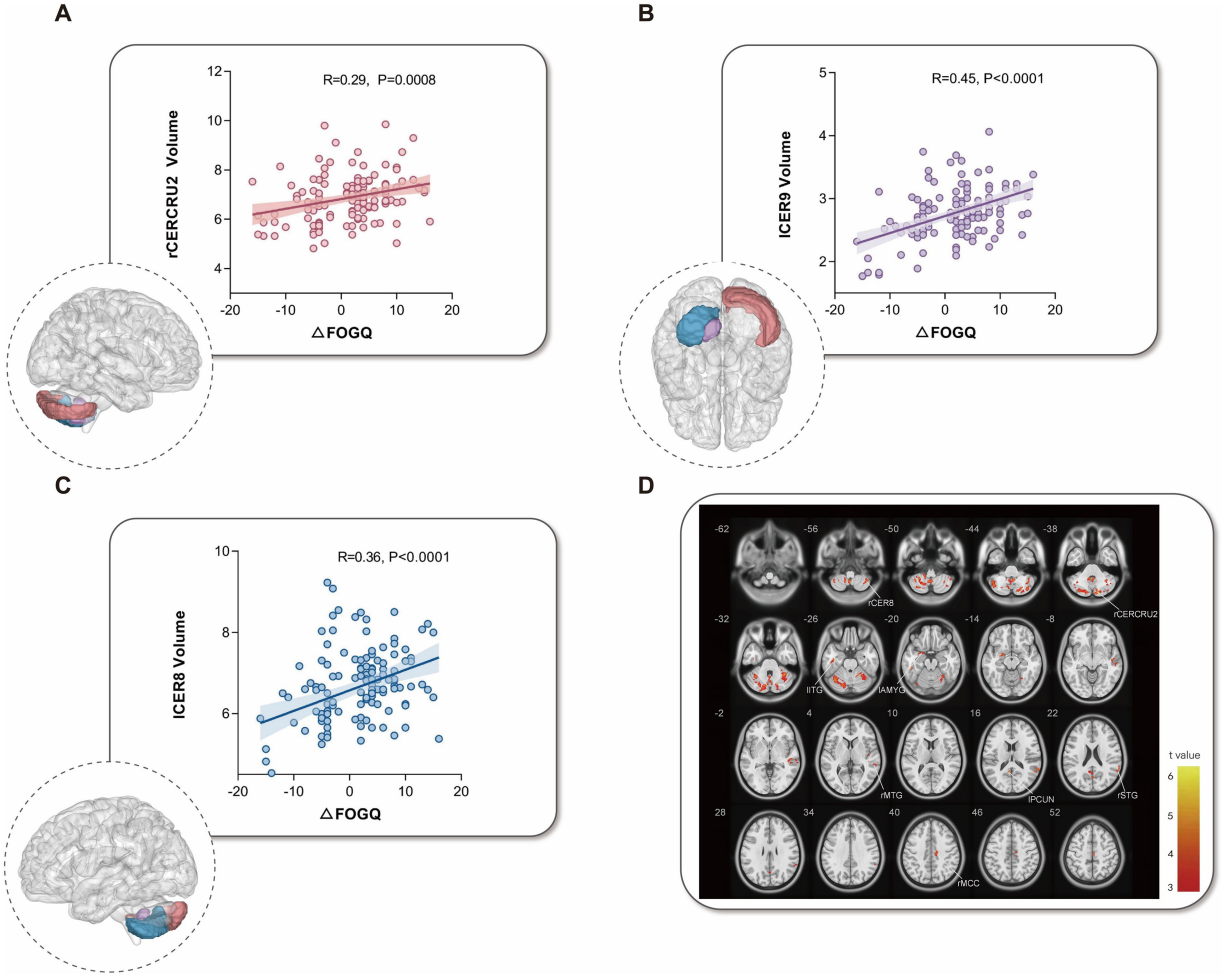
Cerebellar gray-matter volume alterations associated with postoperative freezing of gait outcomes. **(A–C)**: Scatter plots showing the associations between changes in Freezing of Gait Questionnaire scores (ΔFOGQ = Pre_score_-Post_score_; higher values indicate more favorable postoperative outcomes) and gray matter volume (GMV) in **(A)** right cerebellar Crus II (rCERCRU2), **(B)** left cerebellar lobule 9 (lCER9), and **(C)** left cerebellar lobule 8 (lCER8). Insets illustrate the anatomical locations of the corresponding cerebellar regions. **(D)** Voxel-based morphometry (VBM) map of brain regions in which GMV was significantly higher in the no-deterioration group than in the deterioration group. Red clusters indicate regions surviving Gaussian random field (GRF) correction (voxel-wise *p* < 0.001, cluster-level *p* < 0.05).

After adjustment for age, sex, and TIV, patients in the FOGQ deterioration group showed significantly reduced gray matter volume in the left precuneus (Precuneus_L) and right mid-cingulate gyrus (Cingulate_Mid_R) compared with the non-deterioration group (GRF-corrected, voxel-level *p* < 0.001; cluster-level *p* < 0.05, two-tailed). In addition, several cerebellar regions exhibited significantly lower gray matter volume in the deterioration group (GRF-corrected, voxel-level *p* < 0.001; cluster-level p < 0.05, two-tailed) (Figure 2D). These results indicate distinct neuroanatomical alterations associated with postoperative FOGQ worsening. Full statistical details are provided in Supplementary Table 1.

Gray matter volume in the left cerebellar lobule IX (lCER9) was positively correlated with the magnitude of postoperative reduction in FOGQ severity (*p* < 0.001, r = 0.45), indicating that larger lCER9 volume was associated with a lower likelihood of FOGQ deterioration. Similarly, greater gray matter volumes in the left cerebellar lobule VIII (lCER8; p < 0.001, r = 0.36) and the right cerebellar crus II (rCERCRU2; *p* < 0.001, *r* = 0.29) were significantly associated with greater reductions in FOGQ severity (Figure 2). These findings suggest that preserved structural integrity of specific cerebellar subregions contributes to better postoperative gait outcomes and supports a key role of the cerebellum in the dynamic modulation of FOGQ within cortico-basal ganglia-cerebellar networks.

### 3.4 Selection of radiomics features and construction of radiomics scores

Radiomic features were extracted from manually delineated STN ROIs on preoperative T1- and T2-weighted MRI, with T2 acquired in the axial plane (Section 2.5). Images were resampled to 1 × 1 × 1 mm^3^ isotropic voxels for feature extraction. A total of 944 radiomics features were extracted from these ROIs. Features with ICC < 0.75 were removed as a label-independent, quality-control prefilter to ensure good inter-rater reliability. To avoid information leakage, radiomics feature selection and Rad-score construction were implemented within the nested cross-validation framework. Specifically, for each outer fold, z-score standardization, minimum redundancy maximum relevance (mRMR) filtering (top 100 features), and LASSO fitting were performed within the inner 5-fold CV splits to select *λ*. The LASSO coefficient paths and the inner cross-validation deviance curves used for λ selection are shown in Supplementary Figure 3. The finalized pipeline was then refitted on the full outer training set and applied to the held-out outer test set to generate an out-of-fold, fold-specific Rad-score. Because the number and identity of selected features (and coefficients) could vary across folds, all downstream analyses used the out-of-fold, fold-specific Rad-score generated within each outer fold. After model evaluation, a final radiomics signature was refitted on the full cohort using the same pipeline to obtain a single set of coefficients for reporting and clinical translation only; this refitted signature was not used for unbiased performance estimation or for the feature-importance and SHAP inferences reported in the main results. This final model selected 22 features (8 from T1-weighted and 14 from T2-weighted images), and the Rad-score was computed as a weighted linear combination of the standardized features; the full formula and coefficients are provided in Supplementary methods.

### 3.5 Selection and evaluation of multimodal predictive factors

**Figure 3:**
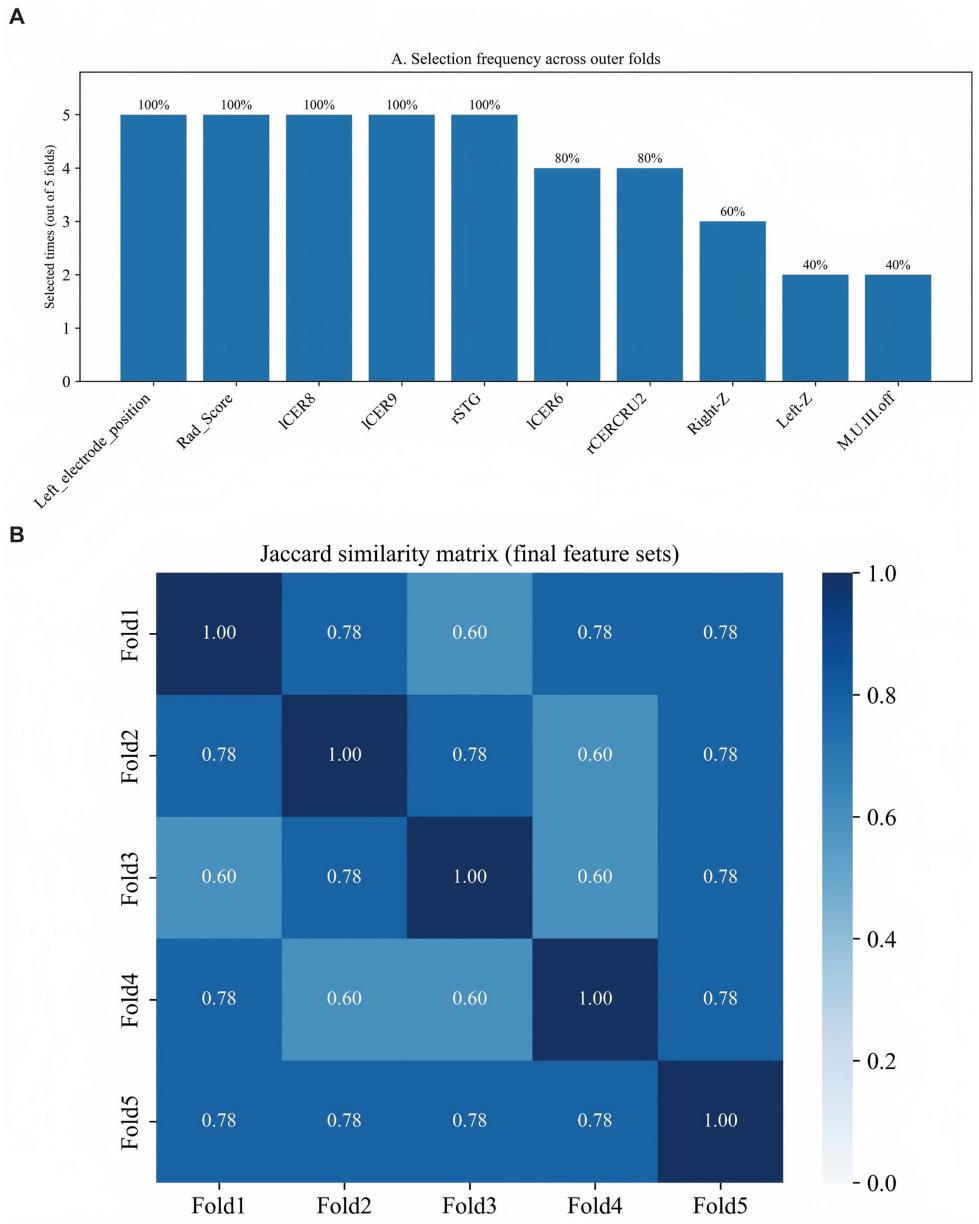
Feature selection frequency and stability across outer folds. **(A)** Selection frequency of each predictor across the five outer folds after the LASSO-Boruta-RFECV pipeline. **(B)** Jaccard similarity matrix of the final feature sets between outer folds.

**Figure 4:**
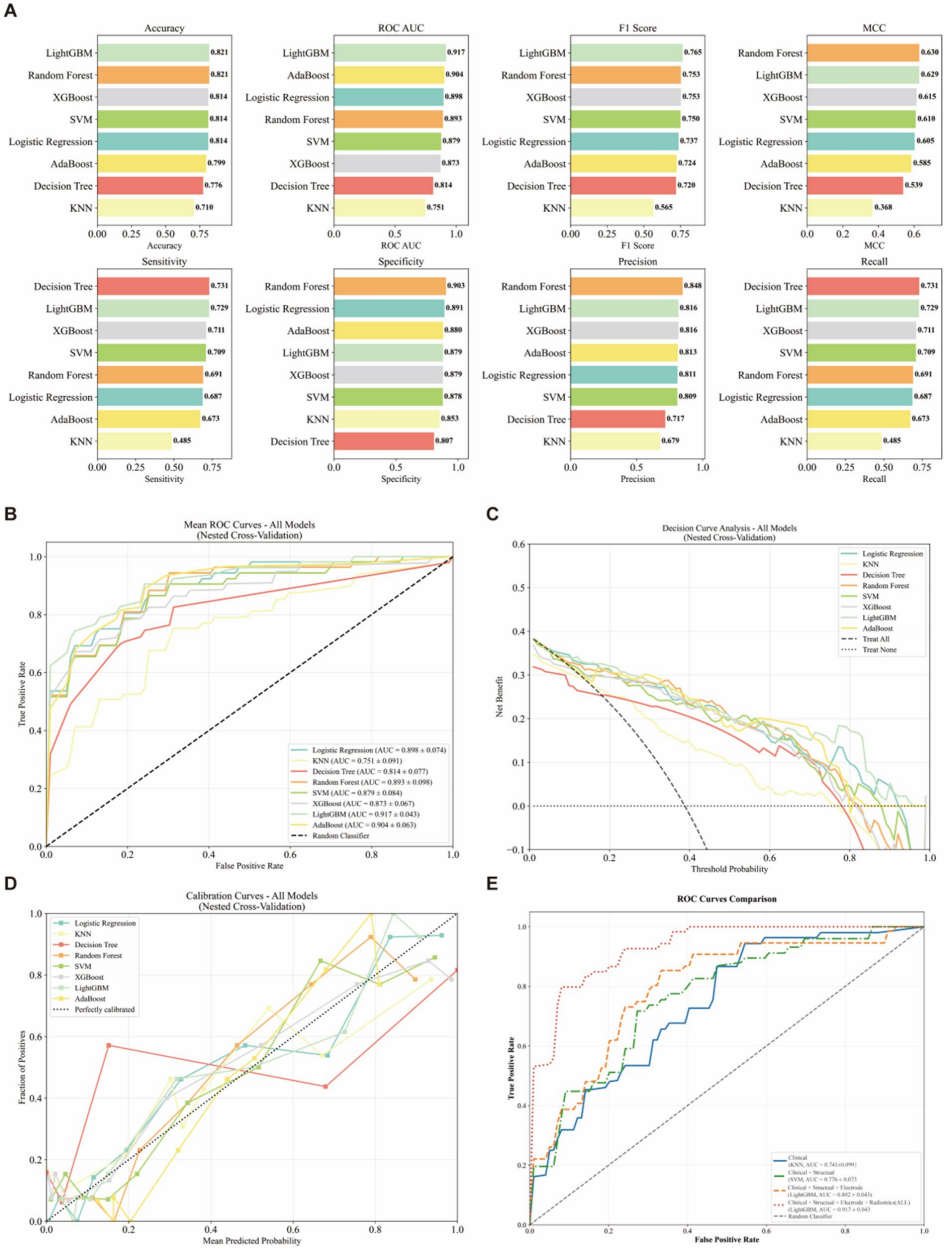
Performance comparison of eight classifiers using 5-fold nested cross-validation. **(A)** Performance comparison of eight classifiers based on outer-loop predictions from 5-fold nested cross-validation, shown as horizontal bar plots for accuracy, ROC-AUC, F1-score, MCC, sensitivity, specificity, precision, and recall (values are means across outer folds). **(B)** Mean ROC curves for all classifiers from 5-fold nested cross-validation. **(C)** Decision curve analysis (DCA) for all classifiers using outer-loop predictions from 5-fold nested cross-validation. **(D)** Calibration curves for all classifiers based on outer-loop predictions from 5-fold nested cross-validation; the dotted diagonal line indicates perfect calibration. **(E)** Incremental ROC curves across feature sets under 5-fold nested cross-validation.

Using a nested feature-selection pipeline (LASSO, Boruta, and RFECV) within the 5-fold nested CV framework, we integrated clinical variables, VBM-derived structural measures, electrode-location features, and the STN Rad-score, selecting up to eight predictors within each outer training fold. The selection frequency and stability across folds are summarized in Figure 3. Eight supervised classifiers (logistic regression, KNN, decision tree, random forest, SVM, XGBoost, LightGBM, and AdaBoost) were evaluated using outer-loop predictions from 5-fold nested cross-validation (Figure 4A). LightGBM showed the best overall performance and was selected as the final model (mean ROC-AUC = 0.917 ± 0.043, bootstrap 95% CI: 0.862–0.963); detailed performance metrics for all models are provided in Supplementary Table 2. The average confusion matrix of the final LightGBM model (outer-loop predictions) indicated high specificity and reasonable sensitivity (TNR = 0.89, TPR = 0.73), consistent with the discrimination metrics (Supplementary Figure 4). Mean ROC curves for all classifiers are shown in Figure 4B. Decision curve analysis suggested that model-guided intensified follow-up and gait-focused care would provide greater net benefit than treat-all or treat-none strategies across clinically relevant thresholds (Figure 4C), and calibration curves showed overall acceptable agreement between predicted and observed probabilities (Figure 4D). For interpretability, feature importance and SHAP values were computed in an out-of-fold manner within the nested cross-validation framework. For each outer fold, SHAP values were calculated for the held-out outer test samples using the model trained on the corresponding outer training set. The background and reference distributions used by the explainer were derived from the outer training data. Global feature rankings were obtained by aggregating SHAP values across all outer test folds, such as mean absolute SHAP. In the incremental ROC comparison across feature sets, incorporating STN radiomics in the full multimodal model yielded the highest discrimination compared with clinical-only and intermediate feature sets (Figure 4E).

**Figure 5:**
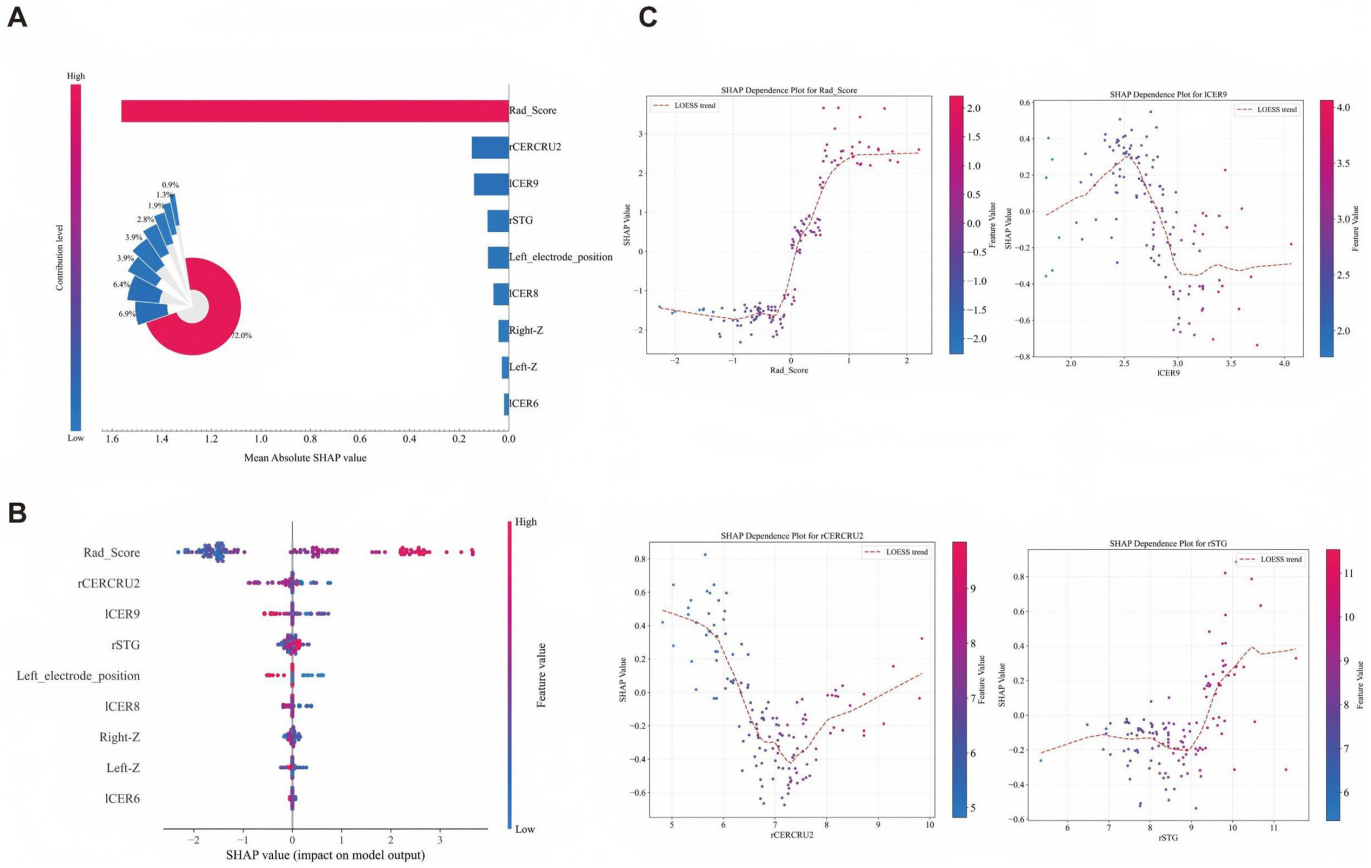
SHAP-based interpretation of the model predicting postoperative changes in FOGQ after STN-DBS. **(A)** Mean absolute SHAP-based global importance (inset: normalized contributions). **(B)** SHAP beeswarm plot showing patient-level effects (color indicates feature value). **(C)** SHAP dependence plots for key predictors (Rad-score, rCERCRU2, lCER9, and rSTG) with LOESS trends.

Based on out-of-fold SHAP summaries aggregated across all outer test folds, the STN Rad-score accounted for 72% of the total feature importance, while structural measures accounted for 20.9% (Figure 5). This distribution highlights the predominant contribution of the STN and shows that combining STN-centered radiomics with whole-brain structural and electrode-localization information improves predictive accuracy and interpretability compared with clinical data alone.

### 3.6 *Post-hoc* analysis of postoperative parameters and FOG outcomes

**Figure 6:**
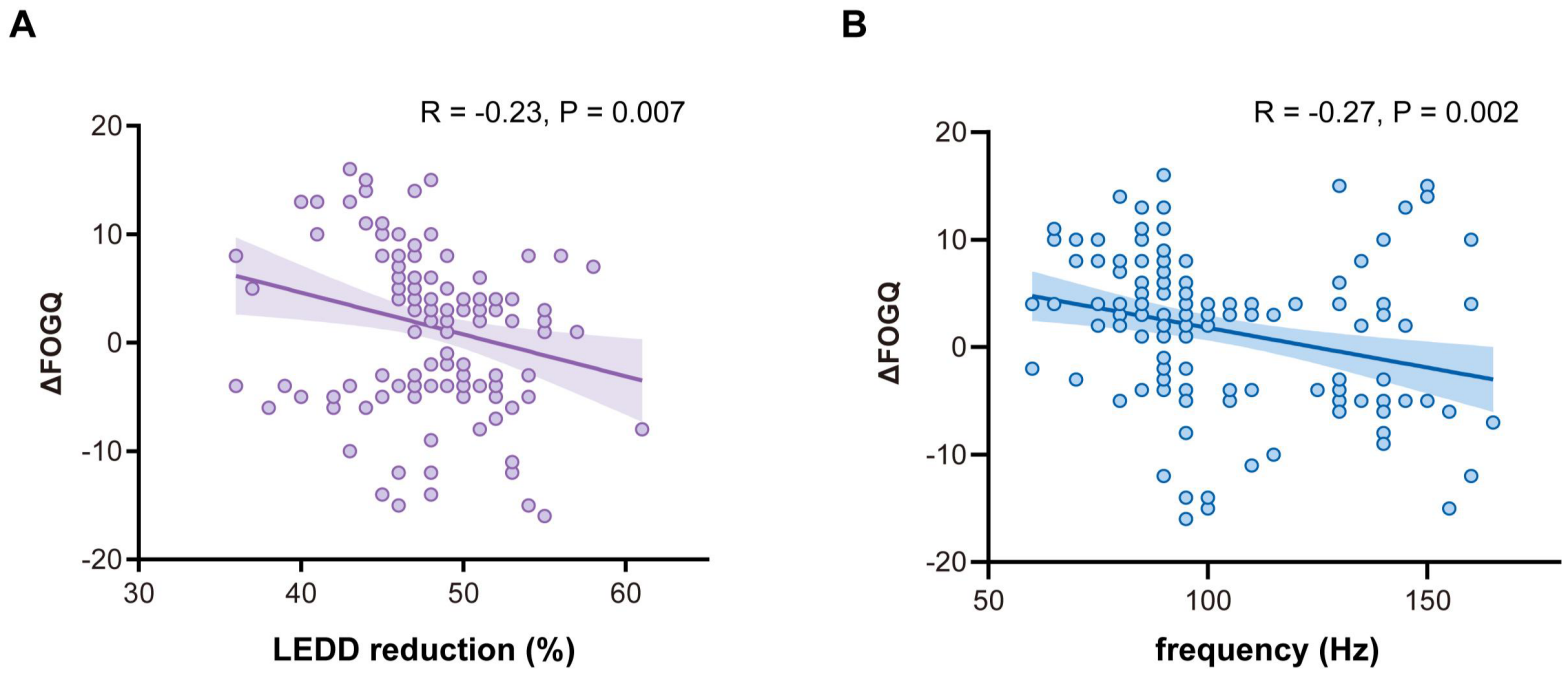
Associations between postoperative parameters and FOG outcomes. **(A)** Relationship between LEDD reduction percentage and ΔFOGQ. **(B)** Relationship between stimulation frequency and ΔFOGQ. ΔFOGQ = Pre_score_—Post_score_; positive values indicate improvement.

To explore whether postoperative management factors were associated with FOG outcomes, additional correlational analyses were conducted. Greater LEDD reduction was associated with worse FOG outcomes (R = −0.23, *p* = 0.007), as was higher stimulation frequency (R = −0.27, *p* = 0.002). These associations are shown in Figure 6.

## 4 Discussion

In this study, we developed and validated a multimodal machine learning model to predict postoperative worsening of FOGQ following STN-DBS, by integrating clinical measures, electrode localization within functional subregions of the STN, voxel-based structural MRI indices, and STN radiomic features derived from T1- and T2-weighted images. Using 5 fold nested cross-validation, model discrimination was evaluated on the held out outer test folds, yielding a mean AUC of about 0.917 for the LightGBM classifier. In this cohort, logistic regression showed that several baseline clinical measures and electrode-location features were associated with postoperative FOGQ outcomes. After adjusting for baseline FOGQ, older age, higher off-medication UPDRS-III scores, and lower MoCA scores were associated with higher odds of deterioration, whereas motor-subregion targeting was associated with lower odds. In the fully adjusted model, only MoCA and left motor-subregion targeting remained significant. At the imaging level, FOGQ deterioration was linked to reduced gray matter volume in the precuneus, mid-cingulate cortex, and specific cerebellar subregions, with STN radiomic signatures contributing substantially to the model’s predictive performance. Together, these findings delineate both large-scale network vulnerability and local STN microstructural determinants of FOG outcomes and provide a rationale for more mechanism-based surgical planning and patient selection.

Previous attempts to predict FOGQ outcomes after STN-DBS are relatively scarce and usually rely on a limited set of clinical variables or coarse whole-brain imaging markers. Although the STN is the principal DBS target, relatively few studies have jointly modeled whole-brain structure, STN microstructure, and electrode localization. By treating the STN both as a radiomic ROI and as a functionally parcellated target, and by integrating these descriptors with clinical risk factors in a single model, we captured FOG-related pathology across multiple levels—from network degeneration and local target configuration to individual susceptibility. Importantly, ablation ROC analysis showed that adding STN radiomics improved discrimination (AUC 0.917 ± 0.043) versus clinical-only (0.741 ± 0.099) and clinical and structural models (0.776 ± 0.073) (Figure 4). This multimodal, mechanism-informed strategy distinguishes our work from earlier single-modality approaches and likely underlies its improved performance in predicting postoperative FOG worsening.

Consistent with the notion that structural brain changes often precede clinical manifestations in neurodegenerative diseases (33), our imaging results provide biological support for the proposed model. Previous work has shown that thalamic structural alterations are closely associated with FOG deterioration (13). Our study extends these observations by identifying gray matter volume loss in several additional nodes, including specific cerebellar subregions, the left precuneus, and the right mid-cingulate gyrus. These changes, distributed across cortical, subcortical, and cerebellar areas, indicate that postoperative FOGQ worsening after STN-DBS does not arise from an isolated focal deficit, but rather from multidimensional network dysfunction encompassing motor, cognitive, and affective circuits.

Among pathways mediating motor and gait control, the cerebellum functions as a key hub. In our cohort, cerebellar subregions showed marked volumetric differences between patients with and without FOGQ worsening, and larger volumes were associated with greater postoperative FOGQ reduction. These findings, together with prior reports, support a central role of cerebellar involvement in FOG pathophysiology (34) and provide a mechanistic rationale for cerebellar-targeted interventions, such as repetitive transcranial magnetic stimulation (rTMS), to modulate cortico-basal ganglia-cerebellar-brainstem loops (35, 36). Beyond predominantly motor pathways, we also observed gray matter loss in the left precuneus and right mid-cingulate gyrus in patients with FOGQ worsening. As hubs for sensory integration, spatial navigation, motor planning, and cognitive-emotional control (37–39), dysfunction in these regions may impair the transformation of sensory cues into coordinated movement and exacerbate gait instability. Taken together, our results support FOG as a distributed network disorder spanning cerebellar-thalamic-cortical and limbic systems (40), rather than a purely motor phenomenon, and may help refine biomarker-based stratification and STN-DBS strategies.

Another noteworthy aspect of this study is that we examined how electrode targeting affects the therapeutic response to STN-DBS. The STN is a key intrinsic glutamatergic projection nucleus within the basal ganglia circuitry and lies at the convergence of the indirect and hyperdirect pathways: it receives direct cortical inputs from motor and prefrontal areas and sends excitatory projections to the GPi and SNr, thereby modulating basal ganglia output and influencing movement initiation, action selection, and gait control (41–44). This network position has led to the STN being regarded as a potential “basal ganglia pacemaker” and implies that stimulation of different functional subregions (motor, associative, limbic) may exert markedly different effects on postoperative gait outcomes (45, 46). Our electrode-localization findings are highly consistent with this concept: contacts located within the motor subregion of the STN, particularly on the left side (with a similar trend on the right), were associated with a significantly reduced risk of postoperative FOGQ worsening after STN-DBS. This is in line with recent work showing that volumes of tissue activated (VTAs) associated with the highest visual analogue scale (VAS) ratings cluster in the dorsolateral STN (47), as well as earlier “sweet-spot” studies based on objective motor scores (48, 49). These results highlight that, during preoperative planning and intraoperative adjustments, the VTA should preferentially encompass the motor subregion, and that postoperative programming should aim to steer current towards motor territories while minimizing spread into associative and limbic subregions. Importantly, we do not imply that motor STN stimulation worsens FOGQ; rather, overall motor improvement can coexist with FOGQ worsening in some patients when stimulation is not sufficiently confined to motor territories or inadvertently recruits non-motor STN networks relevant to gait control, which provides directly actionable guidance for optimizing electrode placement and individualized programming.

At the methodological level, this study has several strengths. We used a well-characterized STN-DBS cohort and a pipeline with 5-fold nested cross-validation and hyperparameter optimization to reduce overfitting and enhance robustness. Radiomic features derived from T1/T2-weighted STN images captured microstructural heterogeneity that is not apparent on conventional MRI and may reflect processes such as neuronal degeneration and gliosis (50–52). By integrating these features with clinical variables, electrode coordinates, and VBM indices, the multimodal model better captured the complexity of FOG-related pathology and outperformed single-modality approaches. Clinically, the primary value of this model lies in its preoperative applicability. By identifying patients at high risk of postoperative FOG worsening before surgery, at a stage when postoperative medication adjustments and stimulation programming have not yet begun, it can inform electrode trajectory planning to prioritize motor subregion coverage, support preoperative patient counseling regarding realistic prognostic expectations, and facilitate early arrangement of gait-focused rehabilitation such as physical therapy or cueing-based interventions for high-risk individuals.

In the exploratory analysis, both LEDD reduction and stimulation frequency were associated with FOG deterioration. The association with LEDD reduction suggests that a dopaminergic (levodopa-responsive) component of FOG may persist in some patients and may become more apparent following substantial postoperative reduction in dopaminergic medication (53). The finding regarding stimulation frequency is in line with previous reports suggesting that lower frequencies may be more favorable for gait in some patients (54). Although effect sizes were small, these results suggest that postoperative management factors may have some bearing on FOG trajectories, and warrant attention in patients identified as high-risk preoperatively. Nonetheless, this study has several limitations. First, it is a single-center retrospective cohort, and the generalizability and stability of the model require further evaluation in external multicenter cohorts within prospective study designs. We also excluded patients with marked disease progression during follow-up (H-Y stage increase >1), which may introduce selection bias and limit generalizability, particularly to rapidly progressing patients. Second, our analyses were based primarily on preoperative structural imaging and clinical data and did not incorporate network-level information from functional imaging or dynamic physiological signals; future work integrating additional modalities such as fMRI, PET, QSM, electrophysiological recordings, and longitudinal imaging may further improve both predictive performance and mechanistic interpretability. Third, postoperative LEDD adjustment and stimulation parameters were not incorporated into the primary model, as the study was intentionally designed as a preoperative prediction tool. Although the exploratory analysis suggests some association between these factors and FOG outcomes, they may represent potential confounders. Prospective studies with systematic postoperative parameter recording are needed to further clarify their role. Future studies incorporating postoperative programming data and medication trajectories would help disentangle the contributions of surgical targeting versus postoperative management to FOG outcomes after STN-DBS. Fourth, the FOG-related outcome was assessed using the FOGQ which, although widely used, may not be sufficiently specific to discrete freezing episodes and could reflect broader gait difficulties. The generalizability of these findings to real-world daily-life FOG in the medication-ON state warrants further investigation; future studies incorporating freezing-specific instruments and objective ambulatory gait monitoring would better capture patients’ real-world gait experience and strengthen the clinical relevance of outcome assessment.

In summary, we developed and internally validated a multimodal machine-learning model that combines clinical measures, electrode localization, structural MRI indices and STN radiomic features to predict postoperative FOGQ worsening in patients with PD undergoing STN-DBS. This approach achieved robust discrimination between patients with and without deterioration and highlighted both network-level vulnerability and STN microstructural alterations associated with adverse gait outcomes. Pending external validation, such models may help refine preoperative risk stratification, inform electrode trajectory planning, and guide early rehabilitation arrangements for high-risk individuals.

## Data Availability

The raw data supporting the conclusions of this article will be made available by the authors, without undue reservation.
