# Supplementary material for: A multimodal machine learning model for predicting postoperative worsening of FOGQ in Parkinson’s disease following STN-DBS
Source: Front Neurol. 2026 May 5;17:1767807. doi: 10.3389/fneur.2026.1767807 (PMC13214268; doi:10.3389/fneur.2026.1767807)
Supplement: Supplementary file 1 [file Supplementary_File_1.docx]

**Supplementary Material for：**

**A multimodal machine learning model for predicting postoperative worsening of FOGQ in Parkinson’s disease following STN-DBS**

**Section 1. Supplementary Methods**

- 1. **Flow diagram of cohort selection**

**
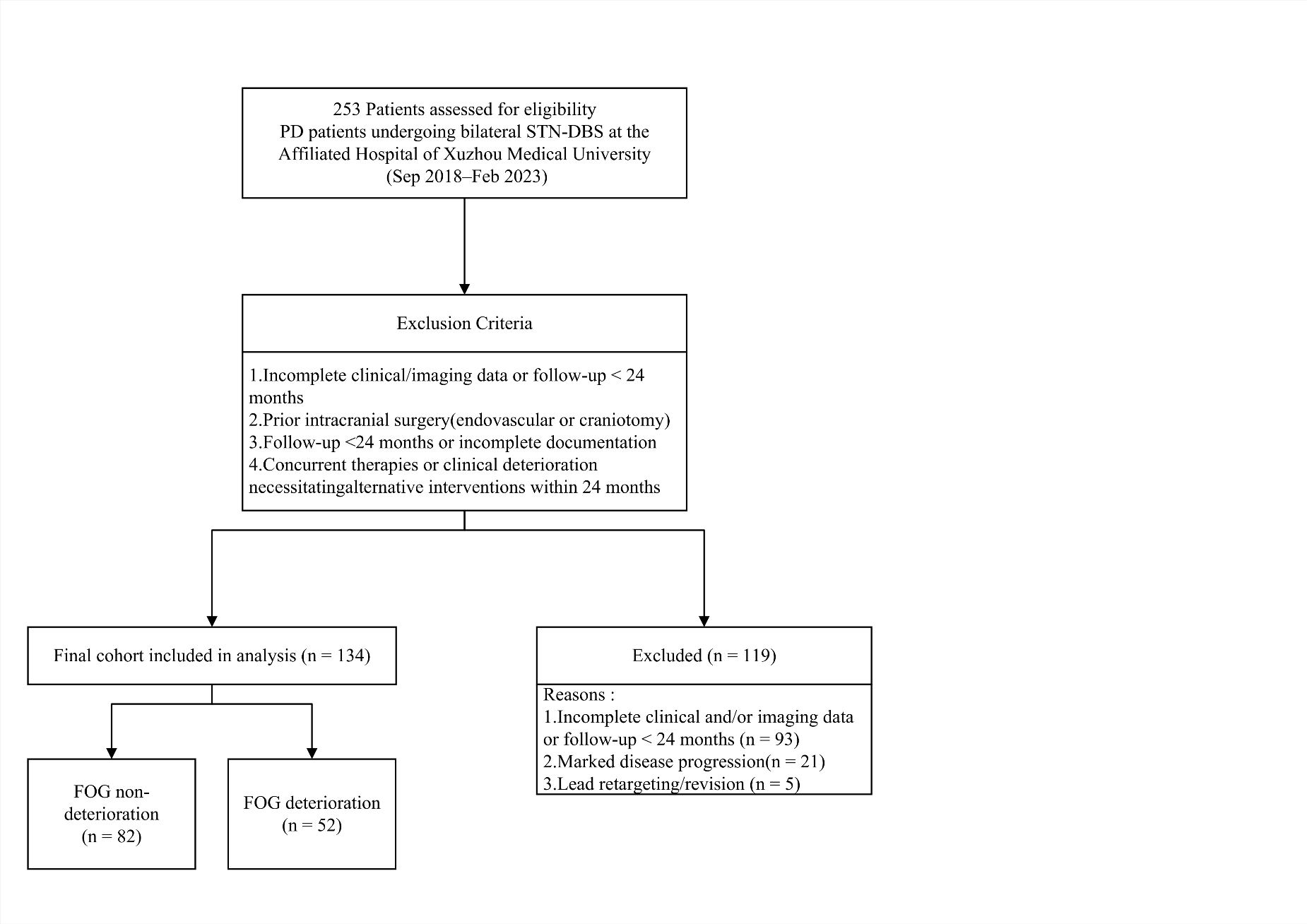
**

**Supplementary Figure 1.** Study flowchart of patient selection and cohort stratification.
A total of 253 patients assessed for eligibility were screened. After applying exclusion criteria, 134 patients with Parkinson’s disease who underwent bilateral STN-DBS and completed ≥24 months of follow-up were included in the final analysis cohort and stratified into FOG deterioration (n = 52) and FOG non-deterioration (n = 82) groups.

- 1. **STN Segmentation and Electrode Targeting**

**
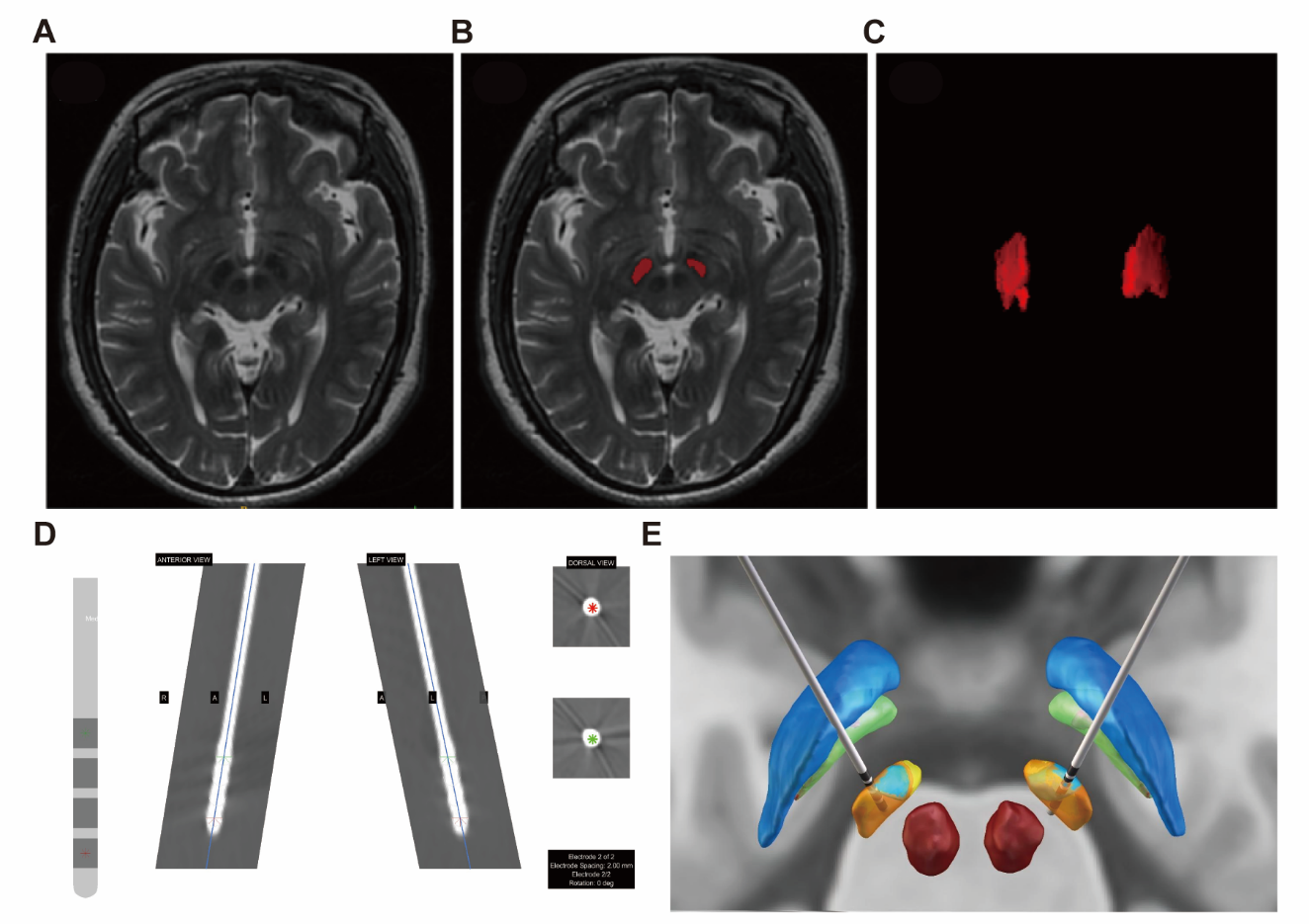
**

**Supplementary Figure 2.** Manual STN segmentation and DBS electrode localization.

A: Preoperative axial T2-weighted MR image. B: Manual segmentation of the bilateral subthalamic nucleus (STN, red) on the T2-weighted image using ITK-SNAP. C: Three-dimensional rendering of the segmented STN masks. D: Three-dimensional reconstruction and localization verification of the DBS electrode contacts. E: Three-dimensional representation of electrode placement relative to the STN subregions—the motor area (orange), associative area (blue), and limbic area (yellow)—illustrating the spatial relationship between activated contacts and each subregion.

**1.3 Radiomics feature selection and Rad-score calculation**

Radiomics feature extraction was performed on manually delineated regions of interest (ROIs) corresponding to the STN on preoperative T1- and T2-weighted MRI. The T2-weighted images were acquired in the axial plane. Prior to feature extraction, images were resampled to 1×1×1 mm³ isotropic voxels. For each STN ROI, a total of 944 radiomics features were initially extracted, including 107 original features, 93 log-sigma–filtered features, and 744 wavelet-filtered features. These features comprised first-order intensity descriptors and higher-order texture metrics (e.g., gray-level co-occurrence matrix [GLCM], gray-level size zone matrix [GLSZM], and neighborhood gray-tone difference matrix [NGTDM] features).

To assess inter-rater reliability and minimize errors arising from manual segmentation, a senior neurosurgeon independently re-delineated the STN ROIs in 30 patients randomly selected from the full cohort of 134 patients. This subset of 30 cases was used exclusively for reproducibility analysis. For each radiomics feature, an intraclass correlation coefficient (ICC) was calculated between the two sets of segmentations. In accordance with commonly accepted standards in radiomics, features with ICC < 0.75 were considered insufficiently reproducible and were excluded from further analysis as a label-independent quality-control (QC) prefilter.

Model development and performance estimation were conducted using a 5-fold nested cross-validation framework. Within each outer training fold, an inner 5-fold CV loop was used to select the LASSO regularization parameter (λ) using the λ_min criterion **Supplementary Figure 3**. For each inner split, z-score normalization, mRMR filtering (top 100 features), and LASSO fitting were performed on the inner-training data only and then applied to the corresponding inner-validation data. After λ was determined, the entire radiomics pipeline was refitted on the full outer training set and applied to the held-out outer test set to generate an out-of-fold, fold-specific Rad-score for downstream modeling.

For reporting and potential clinical translation, we additionally refitted the radiomics pipeline on the full cohort using the same procedure to obtain a single set of coefficients and a reference Rad-score formula. Importantly, this full-cohort refitted model was used for reporting purposes only and was not used for cross-validated performance estimation. The refitted final model selected 22 features (8 T1, 14 T2), and the Rad-score was defined as:

Rad-score = -0.521210

- 0.067012 × t1_wavelet_LHL_firstorder_Kurtosis

- 0.061669 × t1_wavelet_LHH_firstorder_Kurtosis

- 0.024600 × t1_wavelet_HLL_firstorder_Maximum

+ 0.078272 × t1_wavelet_HLH_firstorder_Mean

- 0.000187 × t1_wavelet_HLH_glcm_ClusterProminence

+ 0.083844 × t1_wavelet_HHH_glcm_Idmn

+ 0.121866 × t1_wavelet_HHH_glcm_Idn

- 0.119971 × t1_wavelet_LLL_glcm_Correlation

- 0.013509 × t2_original_firstorder_90Percentile

+ 0.062095 × t2_original_firstorder_Uniformity

+ 0.185315 × t2_original_glszm_LargeAreaHighGrayLevelEmphasis

+ 0.108941 × t2_log_sigma_4_0_mm_3D_firstorder_10Percentile

+ 0.062627 × t2_log_sigma_4_0_mm_3D_firstorder_TotalEnergy

- 0.180765 × t2_wavelet_LLH_firstorder_Maximum

+ 0.042736 × t2_wavelet_HLH_firstorder_Mean

+ 0.068625 × t2_wavelet_HLH_firstorder_Skewness

- 0.125741 × t2_wavelet_HLH_glszm_ZoneEntropy

+ 0.015526 × t2_wavelet_HHL_firstorder_Skewness

+ 0.588679 × t2_wavelet_HHL_glcm_InverseVariance

+ 0.102170 × t2_wavelet_HHL_ngtdm_Contrast

+ 0.133086 × t2_wavelet_HHH_firstorder_Kurtosis

- 0.094777 × t2_wavelet_LLL_glcm_Correlation
In this equation, each term represents the product of a LASSO-derived coefficient and the corresponding z-score–standardized radiomics feature. Feature names follow the standard radiomics convention, indicating the MRI sequence (T1 or T2), filter type (e.g., log-sigma, wavelet), and feature class (e.g., first-order, GLCM, GLSZM, NGTDM). In the main nested-CV analyses, the Rad-score entered into multivariate prediction models was the out-of-fold, fold-specific Rad-score generated within each outer fold as described above (i.e., fitted on the outer training set and applied to the held-out outer test set), thereby preventing information leakage.

**
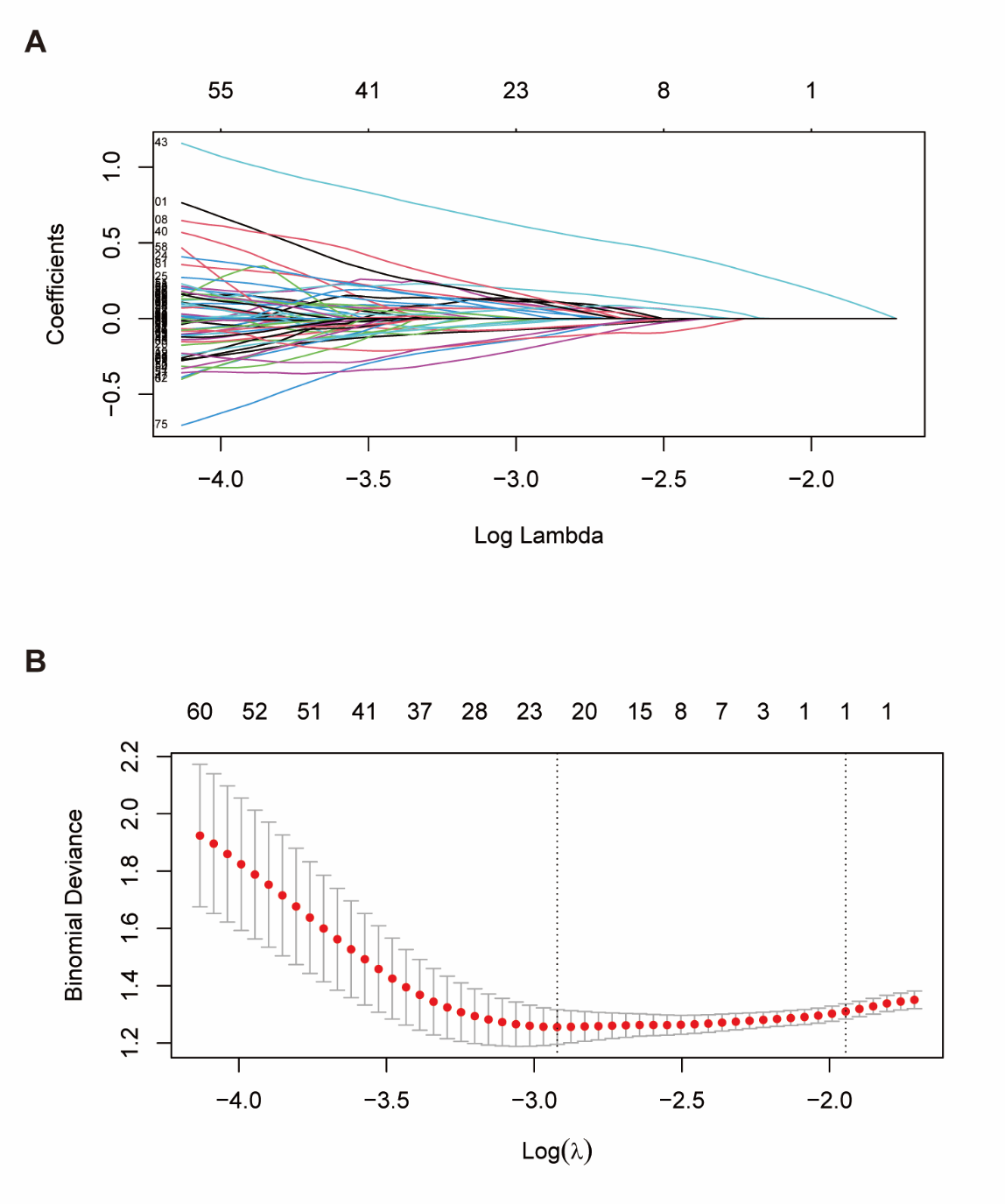
**

**Supplementary Figure 3.** LASSO regression analysis for selection of STN radiomic features.

A: Coefficient profiles of candidate radiomic features plotted against log-transformed λ values. B: Inner 5-fold CV deviance versus log(λ); dashed lines indicate CV-selected candidate λ values. Final Rad-score coefficients were obtained using λ_min.

- 1. **Average Confusion Matrices of All Classifiers**

**
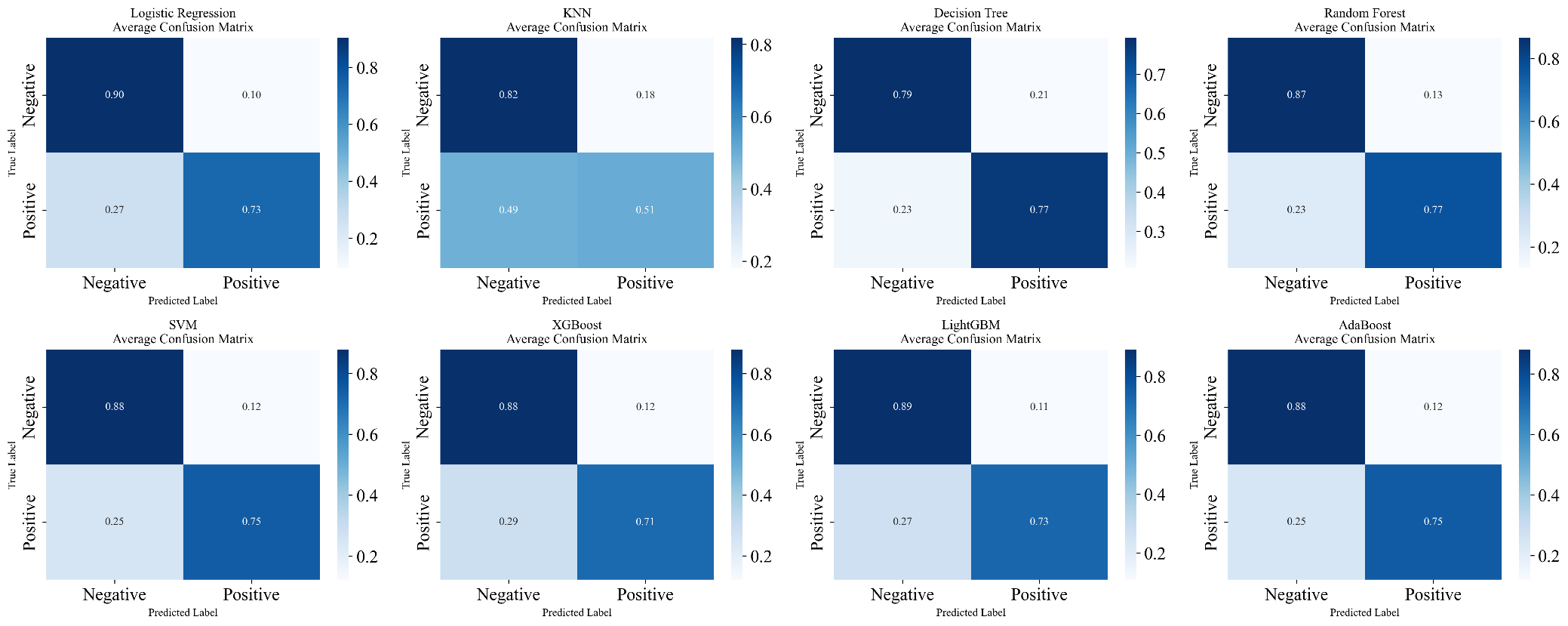
**

**Supplementary Figure 4.** Average confusion matrices for all classifiers under 5-fold nested cross-validation.

Matrices were computed using outer-loop predictions and averaged across the five outer folds. Values represent the mean normalized proportions (rows sum to 1), reflecting sensitivity (TPR) and specificity (TNR) across models.

**Section 2. Supplementary Results**

**2.1 Voxel-based Morphometry (VBM) Analysis Between FOGQ Deterioration and No Deterioration Groups**

| Comparison | Cluster No. | Brain regions | Cluster size | MNI(x,y,z) | | | *t* |
| --- | --- | --- | --- | --- | --- | --- | --- |
|  |  |  |  | x | y | z |  |
| No FOGQ deterioration  vs.  FOGQ deterioration | 1 | Cere8.R | 243 | 40.5 | -52.5 | -54 | 4.45 |
|  |  | Cere7b.R |  |  |  |  |  |
|  |  | CC2.R |  |  |  |  |  |
|  | 2 | CC2.L | 5669 | 18 | -82.5 | -39 | 5.39 |
|  |  | CC2.R |  |  |  |  |  |
|  |  | CC1.L |  |  |  |  |  |
|  |  | Cere6.R |  |  |  |  |  |
|  |  | Cere8.L |  |  |  |  |  |
|  |  | Cere9.L |  |  |  |  |  |
|  | 3 | TPInf.L | 202 | -39 | -13.5 | -28.5 | 4.93 |
|  | 4 | AMY.L | 200 | -18 | 1.5 | -16.5 | 4.58 |
|  | 5 | TPsup.R | 431 | 58.5 | -34.5 | 3 | 4.71 |
|  |  | TPmid.R |  |  |  |  |  |
|  | 6 | TPsup.R | 259 | 64.5 | -46.5 | 19.5 | 4.57 |
|  |  | SMG.R |  |  |  |  |  |
|  | 7 | PCUN.L | 240 | -6 | -52.5 | 15 | 4.83 |
|  | 8 | CGmid.R | 237 | 13.5 | -15 | 42 | 4.51 |
|  |  | SMA.R |  |  |  |  |  |

**Supplementary Table 1.** VBM analysis between patients with and without postoperative FOGQ deterioration.

Note: MNI, Montreal Neurological Institute; x, y, z, are the coordinates of primary peak locations in the MNI space; *t*, statistical value of the peak voxel showing significant VBM differences between the deterioration and non-deterioration groups; Cere8: Cerebellum 8; Cere7b: Cerebellum 7b; CC2: Cerebellum Crus2; CC1: Cerebellum Crus1; Cere6: Cerebellum 6; TPInf: Temporal_Inf; AMY: Amygdala; TPsup: Temporal_Sup; TPmid: Temporal_mid; SMG: SupraMarginal; PCUN: Precuneus; CGmid: Cingulate_Mid; SMA: Supp_Motor_Area; L: Left; R: Right.

**2.2 Performance of Machine Learning Models for Predicting Postoperative FOGQ Deterioration.**

| **Model** | **AUC** | **Accuracy** | **Sensitivity** | **Specificity** | **F1 Score** |
| --- | --- | --- | --- | --- | --- |
| **Logistic Regression** | 0.898±0.074 | 0.814±0.066 | 0.687±0.056 | 0.891±0.031 | 0.737±0.096 |
| **KNN** | 0.751±0.091 | 0.710±0.096 | 0.486±0.124 | 0.853±0.057 | 0.565±0.138 |
| **Decision Tree** | 0.814±0.077 | 0.776±0.081 | 0.731±0.024 | 0.807±0.064 | 0.720±0.097 |
| **Random Forest** | 0.893±0.098 | 0.821±0.054 | 0.691±0.056 | 0.903±0.047 | 0.753±0.054 |
| **SVM** | 0.879±0.084 | 0.814±0.069 | 0.709±0.034 | 0.878±0.067 | 0.750±0.079 |
| **XGBoost** | 0.873±0.067 | 0.814±0.069 | 0.711±0.019 | 0.879±0.056 | 0.753±0.069 |
| **LightGBM** | 0.917±0.043 | 0.821±0.075 | 0.729±0.065 | 0.879±0.076 | 0.765±0.086 |
| **AdaBoost** | 0.904±0.063 | 0.799±0.043 | 0.673±0.043 | 0.880±0.052 | 0.724±0.039 |

**Supplementary Table 2.** Performance comparison of eight machine learning (ML) models

Abbreviations: AUC, area under the receiver operating characteristic curve; MCC, Matthews correlation coefficient; KNN, k-nearest neighbors; SVM, support vector machine; XGBoost, extreme gradient boosting; LightGBM, Light Gradient Boosting Machine.
